# Unveiling a dual mediating chain of cognition-emotion-behavior pathway: How environmental event concerns motivate pro-environmental behaviors among undergraduate students in eastern and central China

**DOI:** 10.3389/fpsyg.2026.1809514

**Published:** 2026-05-20

**Authors:** Wei Zhang, Yanling Chen

**Affiliations:** 1 School of Environmental Science, Nanjing Xiaozhuang University, Nanjing, China; 2 Department of Environmental Science and Engineering, University of Science and Technology of China, Hefei, China

**Keywords:** climate risk awareness, environmental events concerns, environmental responsibility, pro-environmental behaviors, VBN theory

## Abstract

**Introduction:**

The growing global environmental crisis, such as resource depletion and climate change risks, poses a serious threat to the achievement of the Sustainable Development Goals. As the core strategy to alleviate environmental crisis, pro-environment behaviors (PEBs) can significantly reduce carbon emissions and support sustainable development of the environment. However, the influencing factors and underlying mechanisms of PEBs have not yet been fully elucidated.

**Methods:**

A total of 620 undergraduate students from Nanjing, Hefei and Wuhan were selected as the research objects. Correlation analysis, structural equation model, and mediating effect test were employed to delve into the inductive mechanism of environmental events concerns (EEC) of undergraduate students on their PEBs to test the hypothesized paths.

**Results:**

The EEC of undergraduate students directly and positively affected their climate risk awareness, environmental responsibility, and PEBs. Further analysis results demonstrated that EEC also could indirectly strengthened PEBs by the chained mediating pathway of EEC → climate risk awareness → environmental responsibility → PEBs. This uncovered the critical underlying mechanism through which the EEC of undergraduate students influenced PEBs via a dual mediating chain of cognition → emotion → behavior. Additionally, the core bridging role of environmental responsibility and synergy among variables was clarified, which verified the logical chain in Value-belief-norm (VBN) theory.

**Discussion:**

This study is based on the VBN theory and constructs a chained mediating theoretical framework of cognition-emotion-behavior. It clarifies the chained mediating mechanism between EEC and PEBs, supplements the dynamic activation path of the VBN theory, and provides targeted theoretical basis and practical references for promoting the formation of PEBs among college students. Diverse publicity and practical measures should be adopted to enhance the climate risk awareness and environmental responsibility of college students, thereby motivating them to take the initiative to protect the environment.

## 1 Introduction

At present, global environmental problems are becoming increasingly severe. For instance, climate change is exacerbating biodiversity loss and forest depletion. Land desertification and the crisis of freshwater resources directly threaten the foundation of human survival. Meanwhile, marine pollution, chemical and heavy metal pollution, and air pollution erode environmental health from all sides. In recent years, environmental events (such as tropical storms, extreme rainfall, and coastal erosion, etc.) have occurred frequently (Hu et al., 2024), not only weakening the ability of various countries to achieve sustainable development, but also seriously hindering the development process of sustainable societies. Therefore, it is urgent to take action to deal with the above-mentioned environmental problems. Previous studies indicate that pro-environmental behaviors (PEBs) can contribute to the harmony and unity between humanity and nature (Hu et al., 2025), which advances the goal of environmental sustainability. In addition, systematic promotion of PEBs can reduce carbon emissions by 20–30% globally (Gilligan and Vandenbergh, 2020), and PEBs has become a crucial approach to alleviate the environmental crisis.

The influencing factors of PEBs from multiple dimensions such as the individual level, the social level, and the situational level were widely explored. At the individual level, stable psychological characteristics such as gender, educational background, environmental knowledge, and ecological values are the core predictive factors (Casalo and Escario, 2018; Corrado et al., 2022; Wu et al., 2022), while cognitive variables such as climate risk awareness and emotional variables such as environmental anxiety also play a crucial role in PEBs (Clayton et al., 2015; Whitmarsh et al., 2022). At the social level, economic factors (Evans et al., 2013; Asensio and Delmas, 2015), social norms and community relationships (Janmaimool et al., 2026; Tam and Chan, 2018) have complex and even contradictory effects on PEBs, and the synergy between social factors and individual psychology still needs to be further clarified. At the situational level, environmental event experiences, policy background, and information dissemination methods are important dynamic stimulating factors (Steynor et al., 2021; Wang et al., 2025), but existing studies on the relationship between individual attention to specific environmental events and their environmental behaviors is still insufficient. In recent years, the frequent occurrence of environmental incidents has brought the harmonious balance between humanity and nature to a precarious situation. Although the frequency of environmental events may stimulate public awareness of environmental protection (Hoenow et al., 2025), the relationship between environmental events concerns (EEC) and PEBs has not been thoroughly examined.

Recent research has studied the interaction effects between cognitive and emotional mechanisms, among which climate risk awareness (CRA) and environmental responsibility (ER) are regarded as key predictive variables. CRA is defined as an individual’s perception of the climate change threat. ER denotes an individual’s recognition of their own environmental protection obligations. For instance, a positive correlation has been observed between the level of an individual’s CRA and their propensity to endorse climate policies (Clayton et al., 2015). Bamberg and Moeser (2007) confirmed that ER can directly facilitate green consumption by stimulating moral obligations. However, the influence of global environmental events (such as extreme weather, sudden pollution, and the loss of biodiversity) on CRA and ER has not been fully studied yet. The relationship among EEC, CRA, and ER may foster the PEBs of citizens, but the effect of the three on PEBs remains unclear at present. In addition, the interaction relationship among these three factors also requires more in-depth research, and the selection of an appropriate theoretical framework to clarify the interaction mechanism is particularly critical.

Numerous theoretical frameworks have been employed to explain the formation mechanism of environmental PEBs. For instance, the Theory of Planned Behavior (TPB) offers a social cognitive perspective (Ajzen, 1991). This theory predicts behavioral intentions through subjective norms, perceived behavioral control, and attitudes, but lacks support from a value-moral dimension. Moreover, its core structure is not only difficult to precisely match with the variables of this study but also fails to explain how external stimuli like EEC are dynamically transformed into behaviors through multiple psychological variables. In contrast, the Value-Belief-Norm (VBN) theory follows a core logic chain of cognition → emotion/ethics → behavior (Stern, 2000), focusing on the internal psychological transmission mechanism behind PEBs. It is more suitable for exploring how external situational stimuli act on individual psychological factors to influence PEBs. Based on this, this study took college students from Nanjing, Hefei, and Wuhan as the research subjects. The VBN theory was chosen as the basic analytical framework, and EEC was further introduced as an external situational stimulus variable into this framework. CRA was regarded as the specific manifestation of consequence awareness in the VBN theory, and ER was regarded as the integration of responsibility attribution and individual norms. A multi-path theoretical model of EEC driving college students’ PEBs was constructed, and the inductive mechanism of EEC on PEBs was explored. This research aims to provide actionable insights for educators and policymakers, and inspire college students to engage in sustainable development and lead a green future.

## 2 Theoretical background and research hypotheses

### 2.1 Pro-environmental behaviors (PEBs)

PEBs refer to the actions taken to mitigate environmental damage and improve ecological quality (Scannell and Gifford, 2010). Its core essence lies in individuals actively adjusting their behavioral patterns to reduce negative effects on the ecological environment, encompassing both frequent daily behaviors and periodic environmental protection actions, such as energy conservation, waste sorting, and green transportation, etc. It differs from mandatory actions of passively complying with environmental protection regulations, and places greater emphasis on individual subjective will and voluntary actions. However, in daily life, very few people truly practice these environmental protection behaviors. Understanding the reasons for this phenomenon of “knowing but not doing” is of great significance for the promotion of PEBs.

A large body of literature in the fields of psychology and environmental management has studied the factors influencing PEBs (Mi et al., 2021; Lin et al., 2026). These studies approach the issue from multiple dimensions such as individuals, society, and the environment, initially sketching out a framework for the influencing factors of PEBs. However, a systematic and complete mechanism system has not yet been formed. To date, many scholars are still exploring the determinants (Lange, 2023), aiming to solve the problem of the disconnection between environmental protection intentions and behaviors, and providing key insights and empirical support for motivating the public to practice PEBs. For instance, it has been observed that women are more likely to engage in PEB, while people with higher education levels and higher incomes are more inclined to protect the environment (Corrado et al., 2022; Salamah et al., 2026). This disparity essentially stems from the differences in cognitive levels, values, and access to resources among various groups. In addition, environmental event experiences (Steynor et al., 2021; Hoenow et al., 2025), social community relationships (Janmaimool et al., 2026), and climate anxiety (Whitmarsh et al., 2022; Kiss et al., 2026) are also associated with PEBs.

This study aims to explore how EEC, CRA and ER affect PEBs based on the VBN theory and structural equation model. Existing research mostly focuses on the independent effects of individual factors, while insufficient attention has been paid to the interaction relationships and chain mediation mechanisms among the three. This study is precisely based on this research gap, leveraging the quantitative analysis advantages of the structural equation model, to systematically clarify the causal paths among the various variables. These multi-faceted explorations not only enrich the related research on the inductive mechanism of PEBs, but also provide a scientific basis for formulating targeted environmental protection incentive strategies, effectively promoting the formation of sustainable behavioral habits among the public and contributing to the improvement of ecological environment governance efficiency.

### 2.2 Environmental events concerns (EEC)

Currently, environmental incidents are in a high incidence trend, and their threats to ecological security and public well-being are increasingly prominent (Janmaimool and Chudech, 2020). The influence of environmental concerns on PEBs has been increasingly focused in the academic community (Tam and Chan, 2018). Research showed that environmental concerns performed a partial mediating function between self-interest values and PEBs in the private domain (Liu and Wu, 2013). Nevertheless, conventional environmental concern, as a well-documented construct in existing literature, refers to individuals’ long-standing, general, and sustained attention to broad environmental issues such as resource depletion, air pollution, and global warming (Hartmann and Apaolaza-Ibanez, 2012); it is a relatively stable psychological predisposition with no orientation toward specific, targeted environmental incidents. In contrast, this study centers on EEC, which is distinctly different from conventional environmental concern in theoretical connotation. Herein, EEC is conceptualized as the extent of an individual’s situational awareness and affective concern toward concrete, practical environmental incidents including tropical storms, extreme rainfall, and other abrupt ecological events, characterized by remarkable event relevance and timeliness. Rooted in the theoretical interpretation of environmental issues in previous studies, EEC can be regarded as a context- and incident-based subdimension under the overarching concept of environmental concern, representing an in-depth expansion and refinement of the research field. When people actively pay attention to environmental events such as climate change and pollution control, such information not only enhances their ecological awareness, but also stimulates their sense of urgency and willingness to participate in environmental protection, making them more inclined to take practical actions in their daily lives. Therefore, we proposed the following hypotheses:

*H1*: EEC has a positive correlation with PEBs.

The frequent occurrence of extreme environmental events has also intensified public concerns over climate risks. CRA, as one’s subjective perception on the harmfulness, urgency, and vulnerability of climate change, directly affects their environmental protection behavior and policy support. Individuals who have directly experienced extreme climate events have a higher level of risk awareness and adaptability (Broomell and Chapman, 2020). The core logic behind this conclusion is that directly experiencing extreme environmental events enables individuals to more intuitively perceive the severity and urgency of climate change, effectively breaking the sense of distance and indifference towards climate risks, and thereby deepening their understanding of the depth and breadth of climate risks. Furthermore, global catastrophic environmental events can enhance college students’ environmental awareness, thereby strengthening their sense of responsibility for environmental protection (Janmaimool and Chudech, 2020). This further confirms the driving effect of environmental event-related cognition on individuals’ environmental protection psychology and behavior. Therefore, we hypothesized that:

*H2*: EEC exhibits a positive correlation with CRA level.

*H3*: EEC exhibits a positive correlation with ER.

### 2.3 Climate risk awareness (CRA) and its mediating effects

CRA is defined as one’s subjective assessment of the potential negative effects that climate change may have on themselves and society (O'connor et al., 1999). This cognition is the key psychological motivation that triggers and maintains PEBs. It was found that a strong association exists between an individual’s metacognition of climate change and their PEBs (Zheng et al., 2011). Furthermore, CRA not only enhances individuals’ consciousness of environmental issues, but also serves as a crucial factor in driving them to adopt PEBs, especially in scenarios with high policy support and information accessibility (van Valkengoed et al., 2021; Ma et al., 2021). Multiple empirical studies have also consistently shown that CRA will inspire people’s positive willingness and behavior regarding environmental protection (Hu et al., 2025; Syropoulos and Markowitz, 2022; Tasnim et al., 2023) Thus, we formulated the following hypotheses:

*H4*: CRA exhibits a positive correlation with PEBs.

Masud et al. found that as long as people fully recognize the negative effects of turning a blind eye to climate change, they would take active action to protect the environment (Masud et al., 2015). However, CRA only can significantly predict an individual’s attitude towards climate change, but cannot significantly predict PEBs. Fusco et al. conducted an online survey of 448 American undergraduate students and found that CRA was significantly correlated with environmental responsibility behavior (Fusco et al., 2012). Panno et al. showed that CRA played a complete mediating role between cognitive reappraisal and PEBs (Panno et al., 2015). Hence, we proposed the following hypotheses:

*H5*: CRA plays a mediating role between EEC and PEBs.

When people are fully aware of the severe threats brought by climate change and its influence on society, this perception will inspire a strong sense of crisis and a willingness to protect. Realizing that they and their descendants will directly suffer the adverse consequences of climate change prompts them to deeply reflect on their individual responsibilities and transform abstract environmental protection concepts into concrete concerns and commitment to action. CRA is not merely an understanding of the severity of risks, but also involves a profound perception of the correlation and urgency of risks (Vujko et al., 2025). This concrete understanding can break the individual’s sense of detachment from environmental risks, strengthen the awareness that one’s fate is intertwined with that of the environment, and thereby stimulate an inner sense of moral responsibility, motivating the individual to actively assume ER (Magalhaes et al., 2025). Most existing studies have focused on the indirect influence of CRA on PEBs, but have overlooked its direct driving effect on ER, and have not clarified the transmission logic between the two. The clearer and deeper the understanding of climate risks is, the stronger the intrinsic driving force for them to proactively assume environmental protection responsibilities and support sustainable development will be. In view of the foregoing analysis, we put forward the following hypotheses:

*H6*: CRA exhibits a positive correlation with ER.

### 2.4 Environmental responsibility (ER) and its mediating effects

ER refers to an individual’s sense of obligation towards the environment or the responsibility to take action to avoid adverse effects on the environment. Studies showed that ER was instrumental in the preparation for individual participation (Liobikiene and Juknys, 2016; Zareie and Navimipour, 2016). For instance, Clark et al. pointed out that ER urged people to take action for environmental protection (Clark et al., 2003). Likewise, an individual’s sense of responsibility directly influenced their protective intentions (Zhu et al., 2019). That is to say, ER will deeply internalize people’s obligation to protect the environment and directly affect their daily choices and actions. For instance, ER may prompt them to proactively reduce resource consumption (such as water and electricity conservation), adhere to garbage sorting, give priority to green travel methods, and actively participate in environmental protection volunteer activities. Given the analysis presented above, we proposed the following hypotheses:

*H7*: ER exhibits a positive correlation with PEBs.

In addition, people’s active understanding and discussion of environmental events such as climate change and biodiversity loss can profoundly enhance their cognitive level, stimulate their sense of crisis and ethical reflection. This kind of attention prompts the public to examine the influence of their own behaviors on the environment, transform their cognition into an attitude of valuing the environment, and implement it in specific environmental protection actions (Moroni and Dotti Sani, 2024). The focus on EEC is not the primary factor directly driving individual PEBs. Instead, it requires the realization effect transmitted through internal psychological variables, guiding individuals to deeply understand their own obligations as the main actors in environmental governance, and gradually form and strengthen ER (Maggi et al., 2026). ER serves as a crucial link connecting EEC and PEBs, transforming individuals’ external attention to environmental issues into an internal driving force for actively practicing environmental protection actions. Existing studies have preliminarily demonstrated the positive effect of EEC on PEBs. However, most of these studies have overlooked the mediating role of ER. The specific pathways between the two have not yet been clearly defined. Therefore, EEC may be an important driving force for the formation and deepening of ER, which further mediates the influence of EEC on individual PEBs. Thus, we put forward the following hypotheses:

*H8*: ER plays a mediating role between EEC and PEBs.

### 2.5 Theoretical framework

The classic VBN theory encompasses six core constructs: values, ecological worldview/beliefs, awareness of consequences, ascription of responsibility, personal norms, and behavior (Stern, 2000). The core transmission path is consequence awareness → responsibility attribution → personal norms → pro-environmental behavior, representing the cognitive—emotional—behavioral chain, which has been verified by numerous subsequent empirical studies as the core mechanism explaining the internal psychological motive behind PEBs (Fornara et al., 2016; Liobikiene and Juknys, 2016). Noting that the classic VBN theory mainly focuses on the static influence of stable internal psychological variables such as personal norms on PEBs. However, the dynamic activation mechanism of its cognitive-emotional-behavioral core logic chain under contextual external stimuli remains to be further studied. This study focused on college students in the eastern and central regions of China. Based on the core logical chain of the VBN theory, a multi-path theoretical model was constructed to explore the relationship between EEC and PEBs among college students. This model consists of one direct path (EEC → PEBs) and three chain-mediated indirect paths (EEC → CRA → PEBs, EEC → ER → PEBs, and EEC → CRA → ER → PEBs). The EEC → CRA → PEBs path reflects the cognition → behavior chain of VBN theory, the EEC → ER → PEBs path corresponds to the emotion/ethics → behavior chain, and the EEC → CRA → ER → PEBs path aligns with the complete cognition → emotion/ethics → behavior chain of VBN theory.

To focus on exploring the dynamic driving mechanism of contextualized EEC on the PEBs of college students, and to avoid variable redundancy, this study did not include stable internal psychological variables such as values and ecological worldviews, which have been fully confirmed by existing research. Instead, it involves designing specific variables based on the research context and the characteristics of university student. CRA is set as a mediating variable at the cognitive level and is a contextualized refinement of the consequence awareness in the VBN theory, specifically reflecting the subjective perception of individuals regarding the potential negative effects that climate change may bring (O'connor et al., 1999). ER is set as an intermediate variable at the emotional/normative level. It integrates the core connotations of responsibility attribution and personal norms in the VBN theory. PEBs serve as the outcome variable in this study and are in line with the core dependent variable of the classic VBN theory. They represent the final behavioral output of the cognitive-affective psychological transmission chain. EEC is designed to study newly added contextual external stimulus variables, which possess significant event correlation and timeliness. EEC is also an external triggering factor that activates the internal cognitive - emotional - behavioral chain of college students. The core logical chain EEC → CRA → ER → PEBs constructed in this study is highly consistent with the cognitive-emotional-behavioral core of the VBN theory. This is also the core theoretical basis for using VBN theory as the basic analytical framework in this study.

**Figure 1:**
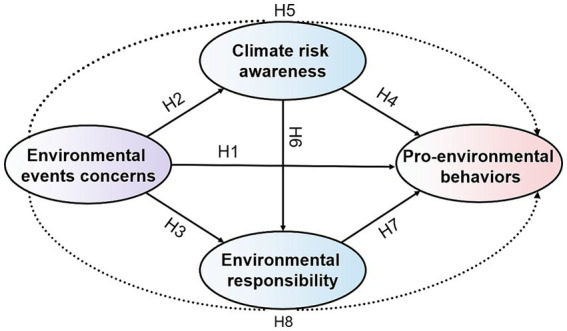
Conceptual diagram of the theoretical model.

Based on the relationships among the variables in the aforementioned assumptions, a conceptual model of the influence paths for college students’ PEBs was constructed and illustrated in Figure 1.

## 3 Methodology

### 3.1 Questionnaire design

To confirm the research hypothesis of the conceptual model on the influencing factors of PEBs, a questionnaire with undergraduate students as the research subjects was designed, aiming to clarify the current situation of PEBs among undergraduate students and verify its influencing factors. The dimensions of EEC, CRA, ER, and PEBs in the questionnaire successively referred to the scales designed by Dunlap et al. (2000), Leiserowitz (2006), Janmaimool and Chudech (2020), and Kaiser and Wilson (2000), and were modified according to the requirements of this study. All the scales adopted the five-point Likert scale. The environmental incident concern scale, from low to high, indicated from “none at all” to “very well.” The other dimensions, from low to high, represent the change from “completely disagree” to “fully agree.” The questionnaire first underwent a pre-survey. Based on the pre-survey data and feedback from the research participants, a series of revisions of the questionnaire were undertaken to improve its content and clarity, and ultimately the questionnaire in Supplementary Table S1 was formed. During the survey, key demographic characteristics, including gender, age, and professional category of the respondents, were also collected. The original scale was designed in English. To improve the translation quality, this study adopted the back-translation method (Brislin, 1970). Specifically, one researcher translated the questionnaire into Chinese, another researcher translated it back into English, and then, a researcher in the field of environmental psychology analyzed the three versions of the scale, revised and optimized the scale to ensure its equivalence before finalizing the questionnaire.

### 3.2 Data collection

Data collection work was carried out in April 2025, aiming to gain a comprehensive understanding of the status quo and underlying factors of PEBs among undergraduate students. The questionnaire survey was reviewed and approved by the ethics committee of the author’s affiliation. The research employed a stratified random sampling method to select undergraduate students from three provincial capital cities (Nanjing, Hefei, and Wuhan) in the Yangtze River Delta and Central China regions. These cities have similar economic and social development levels, highly concentrated higher education resources, a complete environmental protection policy system, and significant implementation effectiveness, providing a homogeneous regional context for exploring the inductive mechanism of EEC on PEBs in this study. The participants were recruited through stratified random sampling. The specific sampling process was as follows: firstly, the universities in the three cities were stratified according to their types and scale of operation. Six universities (two in each city) were randomly selected based on the principle of equal probability. Secondly, within the selected universities, the levels were further divided according to the discipline categories and the grade of study. The sample size was allocated proportionally based on the total number of students in each discipline and grade, and the students from corresponding classes were randomly selected to ensure that all eligible students within the sampling framework had the same probability of being selected, thus guaranteeing the representativeness of the sample.

The researchers released formal questionnaires through an online platform. All the questionnaires were filled out independently, voluntarily and anonymously by the participants. Throughout the entire answering process, no researchers were present, supervising or intervening. No external inducement or answering pressure was exerted on the participants. The respondents were assured that the data collected would be strictly confined to this study. The questionnaire survey process adhered to ethical standards and effectively protected the personal information of the respondents and the content of the questionnaire. A total of 684 questionnaires were distributed for this survey. After eliminating invalid responses, 620 valid ones were retrieved, yielding an effective rate of 90.64%. After the questionnaires were collected, statistical analyses were performed using SPSS 27.0 software and Amos 28.0 software.

## 4 Results

### 4.1 Demographic profiles of respondents

The characteristics of the data indicated that among the 620 surveyed undergraduate students, 59.03% were urban students and 40.97% were rural students (Table 1). In terms of gender, 47.42% were female and 52.58% were male, with a relatively reasonable gender distribution. In terms of the educational stage of study, the third-year university students were the most numerous, accounting for 27.74%. First-year college students accounted for the smallest proportion of 20.81%. From the perspective of professional categories, students from the disciplines of humanities and social sciences were the largest, accounting for 33.55%. The proportion of science and engineering students ranked second, at 28.55%. The proportion of art students was the smallest, at only 12.09%.

**Table 1:** Characteristics of participants.

Variable	Options	*n*	Percent (%)
Gender	Male	294	47.42
	Female	326	52.58
School year	First year	129	20.81
	Second year	167	26.93
	Third year	172	27.74
	Fourth year	152	24.52
Academic directions	Science and engineering	177	28.55
	Humanities and social sciences	208	33.55
	Economic management category	84	13.55
	Art category	75	12.09
	Other	76	12.26
Location of family residence	Countryside	254	40.97
	City	366	59.03

### 4.2 Reliability and validity test

The reliability and validity of the latent variables in the questionnaire were tested by using SPSS27.0 software. Table 2 exhibits that the Cronbach’s Alpha of all measurement dimensions was greater than 0.7, and the McDonald’s *ω* values of each dimension ranged from 0.855 to 0.895 (all > 0.7), implying that the overall reliability of the questionnaire was excellent. The factor loadings of all measurement items were greater than 0.6, and the combined reliability (CR) of all dimensions was higher than 0.7, suggesting that each observed variable could well reflect each of its corresponding latent variables. The mean variance draw (AVE) of each latent variable was all larger than 0.5, indicating that the questionnaire had superior aggregated validity.

**Table 2:** Results of normality test for descriptive statistics and measurement question items for each dimension.

**Dimension**	**Item**	**Estimate**	**AVE**	**CR**	**Cronbach's Alpha**	**McDonald's ω**	**Kurtosis**	**Skewness**
EEC	EEC1	0.791	0.612	0.826	0.824	0.895	−0.159	−0.301
	EEC2	0.746					0.972	−0.864
	EEC3	0.808					0.124	−0.410
CRA	CRA1	0.812	0.545	0.779	0.766	0.866	0.177	−0.399
	CRA2	0.604					0.366	−0.335
	CRA3	0.792					1.156	−0.661
ER	ER1	0.807	0.543	0.779	0.773	0.869	−0.433	−0.127
	ER2	0.636					−0.856	−0.112
	ER3	0.757					−0.838	−0.212
PEBs	PEBs1	0.676	0.528	0.788	0.788	0.855	−0.160	−0.275
	PEBs2	0.624					−0.458	−0.221
	PEBs3	0.702					0.297	−0.517
	PEBs4	0.600					1.527	−0.709
	PEBs5	0.662					0.356	−0.482

Structural validity was evaluated using exploratory factor analysis. The KMO measure of sampling adequacy was 0.839 (> 0.800), and Bartlett’s test of sphericity was statistically significant (*p* < 0.001), indicating that the questionnaire data was suitable for factor analysis. Using principal component analysis with varimax orthogonal rotation, four principal factors were extracted based on the criterion of eigenvalue > 1, with a cumulative variance explained of 65.02%. All items loaded significantly on their hypothesized factors (factor loadings of 0.649–0.855) without cross-loading (cross-loadings < 0.45), and the extracted factor structure was consistent with the theoretical dimensions of the questionnaire. These results demonstrated that the questionnaire exhibited sound structural validity (Supplementary Table S2).

Since all the variables in this study were derived from the same subject and the same time point’s self-report questionnaire, there is a potential risk of common method bias (CMB). This study employed the common latent factor method (Gkorezis, 2015) and combined with Harman single-factor test to conduct CMB testing. In the baseline model, the common method latent variable was included to construct a corrected model and for comparative analysis. The results showed that the variance explained by the first common factor in the Harman unrotated single-factor test was 32.92% (Supplementary Table S2), which was below the critical threshold of 40%. After including the latent variables, there was no significant improvement in the model fitting indicators (ΔCFI = 0.004, ΔRMSEA = 0.002, ΔSRMR = 0.009), and the core path coefficients did not undergo substantive changes. These results indicated that the CMB in this study was within an acceptable level, and the data was suitable for subsequent hypothesis verification and structural equation model analysis.

Discriminant validity was verified using the criterion that the square root of the AVE of each variable should be larger than the correlation coefficient between that variable and other variables. As shown in Table 3, all the data meet the requirements, confirming the satisfactory discriminant validity of this scale.

**Table 3:** Results of differentiated validity test for each dimension of the PEBs for undergraduate students.

Variant	EEC	CRA	ER	PEBs
EEC	0.782			
CRA	0.176	0.738		
ER	0.221	0.400	0.737	
PEBs	0.341	0.405	0.374	0.728

The diagonal numbers were the square roots of AVE.

### 4.3 Normality test

The univariate normality of each PEBs measurement item for undergraduate students was tested using skewness and kurtosis statistics. Based on the criterion put forward by Kline (Ginestet, 2006), if the absolute value of the skew coefficient was less than 3 and the absolute value of the kurtosis coefficient was less than 8, the data was considered to be close to a normal distribution. Table 2 exhibits that the absolute values of the skew coefficient and kurtosis coefficient of each measurement item in this study were all within the standard range. Thus, it could be considered that the data of each measurement item in the PEBs questionnaire for undergraduate students approximately followed a normal distribution.

### 4.4 Correlation analysis

The influence of EECs on PEBs of undergraduate students was analyzed using SPSS 27.0 software, and the correlations among various variables were studied through Pearson correlation analysis (Table 4). There was a clear correlation among the various variables. The correlation coefficients between EEC and CRA, ER, and PEBs were 0.176, 0.221, and 0.341, respectively. The correlation coefficients between CRA and ER and PEB were 0.400 and 0.405, respectively. The correlation coefficient between ER and PEB was 0.374. And they were all significant at the 99% level. As evidenced by the above analysis, significantly positive correlations among the four variables of EEC, CRA, ER, and PEBs in this study was confirmed. It was necessary to utilize the structural equation model to further investigate their influence paths.

**Table 4:** Results of Pearson correlation analysis between dimensions.

Dimension	EEC	CRA	ER	PEBs
EEC	1			
CRA	0.176**	1		
ER	0.221**	0.400**	1	
PEBs	0.341**	0.405**	0.374**	1

^*^p < 0.05, ^**^p < 0.01, ^***^p < 0.001.

### 4.5 Structural equation model analysis

#### 4.5.1 Model fitting test

This study employed Amos 28.0 software for model fitting and validation. To verify the rationality and superiority of the hypothesized model, a non-mediation model (M1) was constructed (only considering the direct paths of EEC → PEBs, EEC → CRA, and EEC → ER, excluding all mediating paths of CRA and ER). The hypothesized chain mediation model was denoted as M2 (EEC → CRA → ER → PEBs). The fitting results of the two models were presented in Table 5. The results showed that the GFI, AGFI, TLI, CFI, and NFI of both models were greater than 0.9, reaching the ideal fitting level (Zhou et al., 2023), indicating that the relationship framework among the variables in this study was fundamentally reasonable. By comparing the core fitting indicators of M1 and M2, it was found that the fitting performance of M2 was significantly better than that of M1. Specifically, the CMIN/DF value of M2 was significantly lower, reflecting a better fit with the actual data, and the lower RMSEA value also indicated a better absolute fit. Therefore, this study selected the chain mediation model for subsequent analysis.

**Table 5:** Model fit test.

Norm	Non-mediation (M1)	Actual results (M2)	Accept value
CMIN/DF	2.219	1.599	<3
RMSEA	0.044	0.031	<0.05
GFI	0.973	0.974	>0.9
AGFI	0.968	0.962	>0.9
TLI	0.978	0.981	>0.9
CFI	0.985	0.985	>0.9
NFI	0.973	0.961	>0.9

#### 4.5.2 Simulated path verification

**Figure 2:**
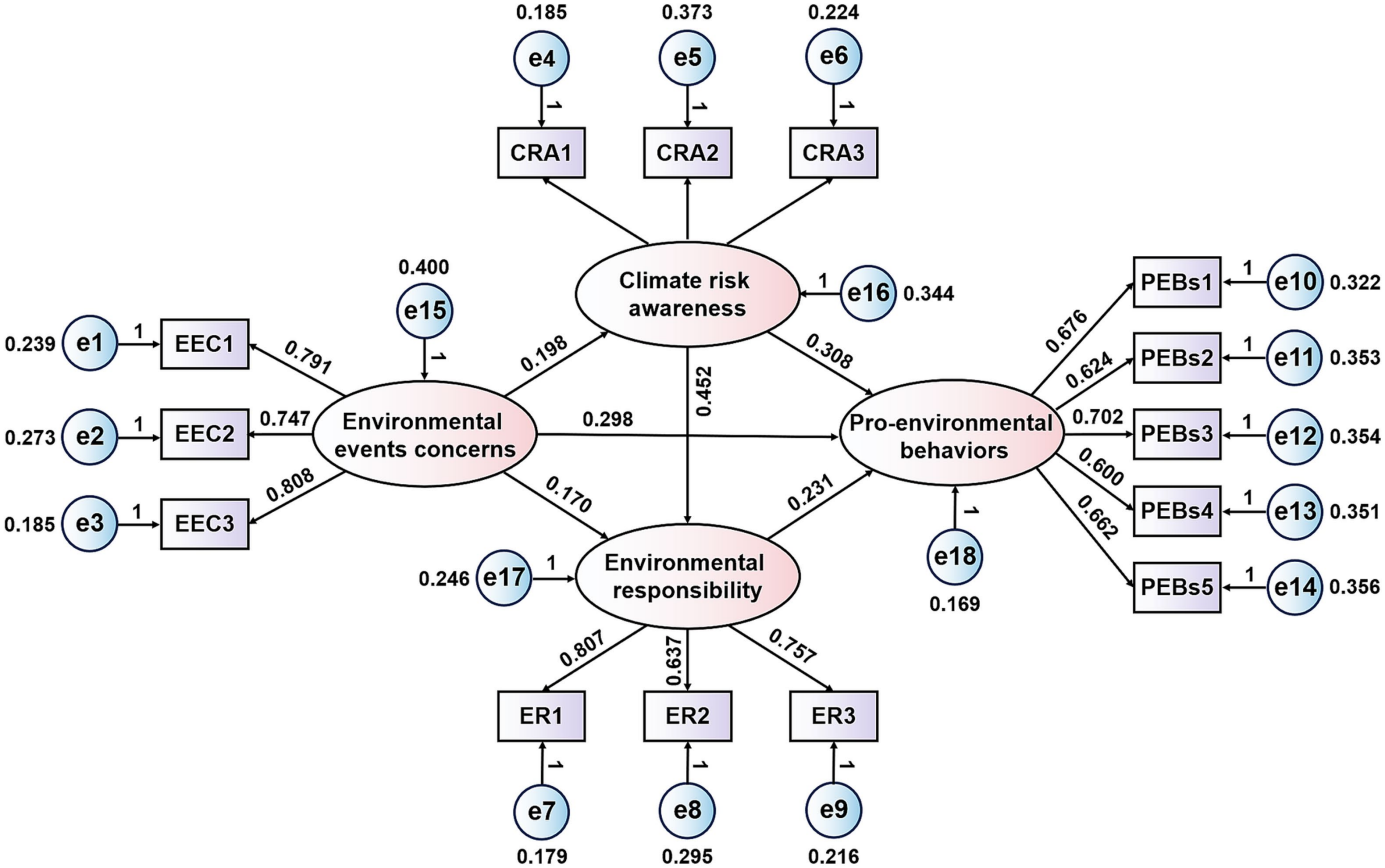
Influence path of the structural equation model of undergraduate students’ PEBs.

Table 6 shows that the path coefficients of the effect of environmental event concern on undergraduate students’ PEBs, CRA and ER were 0.298, 0.198 and 0.170 respectively, and EEC had a significant positive correlation with undergraduate students’ PEBs, CRA, and ER (*p* < 0.001). Therefore, hypotheses H1, H2 and H3 were confirmed. The path coefficients of the effect of CRA on undergraduate students’ PEBs and ER were 0.308 and 0.452 respectively, and CRA had a significant positive correlation with both undergraduate students’ PEBs and ER (*p* < 0.001). Hypotheses H4 and H6 held true. In addition, ER also had a significant positive correlation with on undergraduate students’ PEBs (path coefficient was 0.231, *p* < 0.001), and hypothesis H7 was true. The above results confirmed that the cognitive-emotional-behavioral chain constructed in this study is statistically valid, and this chain is consistent with the core logical chain of the VBN theory. The influence path of the structural equation model of undergraduate students’ PEBs was shown in Figure 2.

**Table 6:** Results of hypothesis testing of path relationship of structural equation model for undergraduate students’ PEBs.

Path relationship	Estimate	S.E.	C.R.	*p*
EEC → PEBs	0.298	0.040	6.190	***
EEC → CRA	0.198	0.047	4.002	***
EEC → ER	0.170	0.043	3.615	***
CRA → PEBs	0.308	0.049	5.527	***
CRA → ER	0.452	0.050	8.766	***
ER → PEBs	0.231	0.050	4.123	***

^***^p < 0.001.

### 4.6 Mediating effect test

To further elucidate the interaction mechanism between EEC and PEBs, this study examined the significance and effect size of each mediating path. The results in Table 7 indicated that all three mediating paths passed the significance test (*p* < 0.01), and the 95% confidence intervals did not include 0, suggesting that each mediating path was statistically significant. This further indicated that EEC can indirectly influence PEBs through different mediating chains.

**Table 7:** Hypothesis testing of mediating effects.

Intermediary path	Standard effect coefficient(β)	95% CI		*p*
		Lower limit	Upper limit	
EEC → CRA → PEBs	0.053	0.026	0.085	***
EEC → ER → PEBs	0.033	0.010	0.060	**
EEC → CRA → ER → PEBs	0.023	0.010	0.039	**

**p < 0.01, ***p < 0.001.

Specifically, in the “EEC → CRA → PEBs” path, the standardized effect coefficient *β* of this path was 0.053 (*p* < 0.001), with a 95% confidence interval of [0.026, 0.085]. This indicated that EEC could positively influence students’ cognitive level of climate risks, thereby indirectly promoting the occurrence of their PEBs. Moreover, the indirect effect of this path was significant, proving the validity of H5. In the “EEC → ER → PEBs” path, the standardized effect coefficient *β* = 0.033 (*p* < 0.01), with a 95% confidence interval of [0.010, 0.060], indicated that EEC could indirectly facilitate public environmental protection behaviors by enhancing individuals’ environmental responsibility awareness. The significant indirect effect also confirmed that H8 was supported. The *β* of the serial mediating pathway “EEC → CRA → ER → PEBs” was 0.023 (*p* < 0.01), with a 95% confidence interval of [0.010, 0.039]. Although the standard effect coefficients of the three mediating paths were relatively small, they all passed the significance test, which was in line with the actual driving laws of PEBs. The marginal effect of a single contextual variable typically shows a “weak but significant” characteristic (Meng et al., 2023). Moreover, college students’ PEBs have not yet been firmly established, and there is a transmission loss in the transformation from cognition and responsibility to behavior. Additionally, this group is more likely to take action in response to specific environmental events. This suggested that EEC could first exert a positive influence on CRA, enhancing individuals’ environmental responsibility awareness, and ultimately indirectly promoting PEBs, forming a crucial serial mediating transmission mechanism. Overall, CRA and ER served as critical mediators between EEC and PEBs, which to some extent validates the applicability of the core logical framework of the VBN theory in the context of Chinese college students’ attention to environmental events.

## 5 Discussion and conclusion

### 5.1 Main conclusions

This research employed a structural equation model to test the inductive mechanism and path of EEC on undergraduate students’ PEBs. The main conclusions were as follows.

#### 5.1.1 Direct positive correlation between EEC and PEBs

EEC directly and positively affected the CRA, ER, and PEBs of undergraduate students in this study, which was basically consistent with the research of the international academic community. Brody et al. (2008) found that extreme weather issues could contribute to the formation of public awareness of climate risks. Janmaimool and Chudech (2020) found that the combination of domestic environmental problems and global environmental problems could improve the ER of Thai undergraduate students. This study also confirmed that EEC has a stronger predictive power for Chinese college students’ PEBs than general environmental values, which is consistent with previous research results (Liu and Wu, 2013; Chen and Tung, 2014). The strong predictive power of EEC stems from its concrete and contextual variable attributes. Compared with abstract and stable general environmental values, it can directly break the “psychological distance” of individuals from environmental risks (Gong et al., 2023), quickly activating CRA and ER. This is highly consistent with the “direct positive effect of EEC on PEBs” verified by this study. The formation of this characteristic is closely related to the real-time, visualized and localized dissemination of extreme weather events by social media in recent years. As the primary channel for Chinese college students to obtain environmental information, the concrete dissemination form of social media has enabled environmental risk perception to shift from the cognitive level to the emotional level (Meng et al., 2023; Vrselja et al., 2024), further strengthening the transformation of EEC into PEBs. This dissemination characteristic also highly aligns with the group cognitive behavior characteristics of college students who are easily influenced by concrete stimuli (Li et al., 2022).

#### 5.1.2 Mediating role of CRA and ER

CRA and ER both had a significant positive association with PEBs, and both played a partial mediating role between EEC and PEBs, which were consistent with existing research findings, deepening the understanding of the mediating mechanism of cognitive and affective variables in the process by which EEC influences PEBs. Liu et al. (2026) and Syropoulos and Markowitz (2022) found the cognitive predictive role of CRA on environmental behavioral outcomes. Liu and Wu (2013) also demonstrated that environmental concern can indirectly influence PEBs by enhancing ER, thus laying the empirical foundation for the analysis of the ER mediating effect in this study. Unlike the majority of previous studies that only verified the mediating role of CRA or ER through separate research designs, this study initially confirmed the independent partial mediating effects of both, and basically clarified two parallel transmission paths (EEC → CRA → PEBs and EEC → ER → PEBs) applicable to college students in eastern and central China. Combined with the research conclusion of Scopelliti et al. (2022), the mediating effect of CRA reflected the transformation of external EEC stimuli into individual internal cognitive judgments, while the mediating effect of ER was the elevation of this cognition to emotional and normative awareness. Meanwhile, the significant mediating effect of CRA and ER aligns with the cognitive behavioral characteristics of college students who are sensitive to environmental information and prone to emotional resonance (Li et al., 2022). The quantitative measurement of the mediating effect of the two in this study also provides a basis for subsequent comparative studies on the mediating role across different groups and scenarios.

#### 5.1.3 Synergy among variables

CRA was significantly positively correlated with ER, which validated and expanded the core logical chain of cognition-emotion-behavior in the VBN theory (Stern, 2000), providing new support for the applicability of this theory in the field of climate change and environmental events research. A number of previous studies have verified the validity of this logical chain in scenarios. Latif et al. (2024) and Syropoulos and Markowitz (2022) both confirmed that the synergy between CRA and ER had a significant positive effect on individuals’ pro-environmental attitudes and behaviors. This study expands the application scope of the VBN theory from the influence of stable environmental psychological factors on PEBs to the contextualized EEC effect dimensions. Different from the existing VBN-related studies that focus on the static transmission of stable internal psychological variables (Fornara et al., 2016), this study reveals that EEC can activate individual CRA and further strengthen ER through cognitive judgments of climate risks, forming a chain transmission mechanism of EEC → CRA → ER → PEBs. This work supplements the dynamic activation path of the VBN theoretical logic chain under the stimulation of external environmental events, enriching its explanatory framework and providing a new perspective for the subsequent construction of a multi-dimensional PEBs inductive mechanism model.

### 5.2 Theoretical contribution

This study distinguishes the core content of EEC from the general environmental concerns, enriching the research dimensions of environmental concerns. It supplements the dynamic activation path of the core logical chain of the VBN theory under the stimulation of external environmental events, expanding its application boundary from static internal psychological variables to the contextualized external stimulus domain. Additionally, this work verifies the dual mediating and chain mediating effects of CRA and ER between EEC and PEBs.

The research on EEC is closely aligned with actual conditions, making it highly practical and valuable, and providing a basis for universities and environmental protection departments to formulate targeted measures. In addition, unlike the simple mechanisms of direct influence or single mediator in existing generalized environmental concern research, this study has demonstrated the dual mediating role of CRA and ER, clarified the unique psychological transmission path of EEC affecting PEBs, and this differentiated research finding is an important empirical supplement to the study of the relationship between environmental concern and PEBs. This study provides a new perspective for understanding how EEC affect individuals’ PEBs at the cognitive-emotion-behavioral level.

This study conducts an empirical analysis based on the SEM, expanding the explanatory framework and application boundaries of the VBN theory (Stern, 2000). Previous studies on the VBN theory have mostly focused on stable internal psychological variables such as personal norms and environmental values on the static influence of PEBs (Fornara et al., 2016), and most have adopted a single mediator research design, such as Panno et al. (2015) confirming the mediating role of CRA in the cognition of environmental behaviors, and Liobikiene and Juknys (2016) found that the assumption of responsibility directly influenced people’s environmental behaviors. This study expands the application scope of the core cognitive-emotional-behavioral logic chain of VBN theory from the static psychological dimension to the contextualized EEC domain. It preliminarily verifies the independent mediating effects of CRA and ER, discovers two parallel transmission paths through which EEC influences college students’ PEBs, and reveals the chain transmission mechanism of EEC → CRA → ER → PEBs. This also supplements the dynamic activation path of the VBN theory’s logical chain under external stimuli, and preliminarily validates the applicability of the theory among college students in the eastern and central regions of China.

### 5.3 Practical implications

Given the evidence that EEC played an effective role in promoting PEBs, expanding the channels for the dissemination of environmental incidents may contribute to the implementation of PEBs. For example, ecological and environmental protection departments should promptly notify and follow up on environmental incidents occurring worldwide, thereby drawing college students’ attention to related events. Build a communication matrix for environmental issues by leveraging new media platforms – in collaboration with the new media centers of universities, launch topics such as the Climate Action Program on platforms including Weibo, WeChat, and Tiktok, and push out concrete and localized content on environmental events while abandoning abstract environmental protection slogans. Present extreme climate cases through forms such as data visualization long images and virtual reality scene simulations. The meteorological bureau can utilize new media platforms to carry out live-streaming activities, presenting the process of glacier melting from a satellite perspective to attract college students to participate in the interaction. Universities can invite experts and scholars to hold special popular science lectures to publicize local and global environmental events (such as extreme climate cases and ecological protection achievements), guide college students to concentrate on the real influence of the events, and enhance their awareness of climate risks. In the second classroom, environmental protection activities such as *Model United Nations on Climate Change* and *World Environment Day* environmental event case discussion meeting can be held to effectively enhance students’ personal understanding of environmental events.

In light of the mediating roles played by CRA and ER in promoting PEBs, constructing a cognitive-responsibility-action closed loop may be an effective way to foster PEBs. College teachers serve as a key component in the cultivation of college students’ PEBs, and their educational process will have a subtle influence on students’ growth. Universities should incorporate risk awareness education courses into the important part of teacher training, regularly enrich the intellectual resources of college faculty, and enhance their pro-environment level. Teachers should enrich educational methods in the teaching process and integrate environmental responsibility education into the daily curriculum design. For instance, they can introduce a sustainable development module in general education courses to emphasize individuals’ moral obligations towards the environment. External concerns about environmental events also can be converted into college students’ internal awareness of environmental responsibility through campus environmental protection practices, discussions on environmental issues and other such activities. At the practical education level, responsibility awareness can be transformed into specific PEBs through the formation of group projects or environmental protection volunteer services. Meanwhile, systematic interventions in accordance with the progressive logic of the chain mediating pathway should be conducted, and the effects of each link will be superimposed to offset the issue of small effect size in a single pathway. Targeted environmental education should be delivered in light of the group characteristic of college students who prefer concrete information.

### 5.4 Research limitations and future prospects

There are inevitable shortcomings in our work. Firstly, the sample in this study only includes college students from three provincial capitals in the eastern and central regions of China. The environmental education resources in these areas are superior, which may cause the EEC and PEBs tendencies of the sample to be higher than those in less developed regions. This limits the cross-regional generalization of the research conclusions. In future studies, samples from the western regions and economically underdeveloped areas should be included. Secondly, this study only included undergraduate students from three cities. The research conclusions cannot be generalized to all university students. Future studies can further expand the sample coverage and include samples from different regions and different levels of universities to further test the universality of the research conclusions. Furthermore, the cross-sectional design cannot reveal long-term causal relationships. Subsequent studies need to adopt longitudinal research methods to track the dynamic changes of PEBs. Finally, this study does not conduct in-depth exploration regarding the influence of demographic variables, which constitutes a research direction that is worthy of further investigation. It can be incorporated into the model for conducting robustness tests and group heterogeneity analysis in future studies, which not only helps to verify the universality of the core conclusion but also can further expand the explanatory power and applicable scenarios of the research findings in different groups.

## Data Availability

The raw data supporting the conclusions of this article will be made available by the authors, without undue reservation.
