# Supplementary material for: Unveiling a dual mediating chain of cognition-emotion-behavior pathway: How environmental event concerns motivate pro-environmental behaviors among undergraduate students in eastern and central China
Source: Front Psychol. 2026 May 20;17:1809514. doi: 10.3389/fpsyg.2026.1809514 (PMC13229755; doi:10.3389/fpsyg.2026.1809514)

**Supplementary material for**

**Unveiling a dual mediating chain of cognition-emotion-behavior pathway: How environmental event concerns motivate pro-environmental behaviors among undergraduate students in eastern and central China**

**Wei Zhang^a^, Yanling Chen^b, *^**

*Corresponding author.

*E-mail address:* [chenyanling@ustc.edu.cn](mailto:chenyanling@ustc.edu.cn) (Y. L. Chen)

**Table S1 Variable settings of the questionnaire.**

| **Study variables** | **Measurement items** | **Theoretical support** |
| --- | --- | --- |
| **Environmental Events Concerns**  **(EEC)** | How concerned are you about rising global average temperatures? (EEC1) | 1 = Not at all  5 = Very much  concerned |
|  | How concerned are you about sea level rise? (EEC2) |  |
|  | How concerned are you about the death of aquatic animals due to waste in the oceans? (EEC3) |  |
| **Climate Risk Awareness**  **(CRA)** | Is it very likely that extreme climate disasters will occur in your area in the future? (CRA1) | 1=Completely disagree  5=Completely agree |
|  | Will climate change directly affect your quality of life? (CRA2) |  |
|  | Is global warming an urgent climate crisis? (CRA3) |  |
| **Environmental Responsibility**  **(ER)** | Do you have the responsibility to contribute to reducing carbon emissions? (ER1) | 1=Completely disagree  5=Completely agree |
|  | Is protecting the environment the duty of every citizen? (ER2) |  |
|  | Does the occurrence of environmental events make you realize that protecting the environment is a global responsibility? (ER3) |  |
| **Pro-Environmental Behaviors (PEBs)** | Would you sort garbage in daily life? (PEBs1) | 1=Completely disagree  5=Completely agree |
|  | Would you reduce the use of disposable plastic products in daily life? ( PEBs2) |  |
|  | Would you choose public transportation or shared mobility in daily life? ( PEBs3) |  |
|  | Will you participate in environmental protection initiative activities on campus or in social organizations in daily life? ( PEBs4) |  |
|  | Would you promote environmental protection knowledge to your relatives and friends in daily life? ( PEBs5) |  |

**Table S2** The factor loading matrix after orthogonal rotation.

| **Observed variable** | **Extract components** | | | | **Common factor variance** |
| --- | --- | --- | --- | --- | --- |
|  | **Factor 1** | **Factor 2** | **Factor 3** | **Factor 4** |  |
| EEC1 | – | 0.855 | – | – | 0.755 |
| EEC2 | – | 0.814 | – | – | 0.708 |
| EEC3 | – | 0.852 | – | – | 0.754 |
| CRA1 | – | – | – | 0.851 | 0.738 |
| CRA2 | – | – | – | 0.681 | 0.607 |
| CRA3 | – | – | – | 0.832 | 0.732 |
| ER1 | – | – | 0.824 | – | 0.772 |
| ER2 | – | – | 0.733 | – | 0.546 |
| ER3 | – | – | 0.830 | – | 0.743 |
| PEBs1 | 0.718 | – | – | – | 0.565 |
| PEBs2 | 0.710 | – | – | – | 0.540 |
| PEBs3 | 0.717 | – | – | – | 0.583 |
| PEBs4 | 0.649 | – | – | – | 0.475 |
| PEBs5 | 0.729 |  |  |  | 0.585 |
| Variance explanation rate (before rotation) | 32.392% | 13.625% | 10.281% | 8.721% | – |
| Cumulative variance explained rate  (after rotation) | 19.466% | 35.545% | 50.309% | 65.019% | – |
| KMO | 0.839 | | | | – |
| Bart's spherical value | 2899.489 | | | | – |
| p value | 0.000 | | | | – |


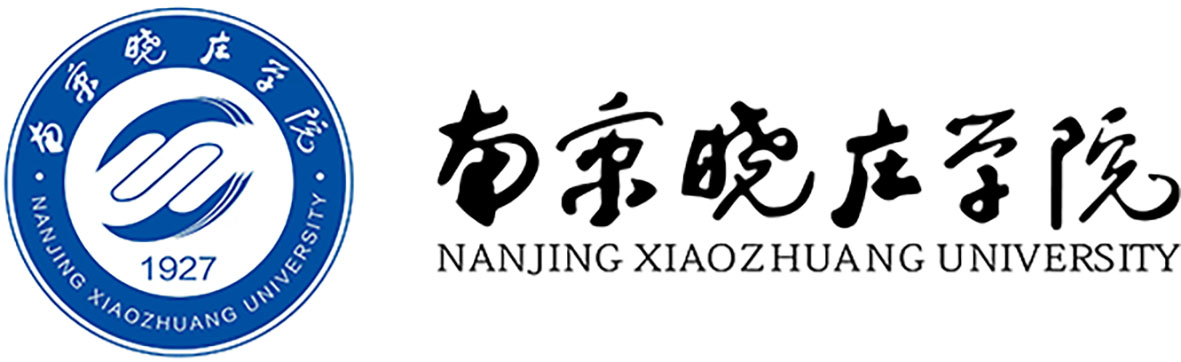
**Informed Consent Form**

Dear participants:

Thank you very much for participating in our questionnaire survey. This is a questionnaire about the influencing factors of college students' pro-environmental behaviors and is only for academic research. During the process of answering the questionnaire, you need to be highly concentrated on the questionnaire materials, carefully observe the content and experience the emotions, make judgments and associations, and keep your brain thinking. Do not let your mind wander or daydream during the scoring process.

This questionnaire survey was reviewed and approved by the ethics committee of the author’s affiliation. This participation is purely voluntary. You can terminate your participation in this research at any time. Such a decision will not result in any adverse consequences. When collecting questionnaire data, personal names and ages are not involved. Only numbers are used to store your information. The collected information will only be used for academic research purposes, and personal data will be kept strictly confidential. If you have any questions about this study, you can send an email to zhangwei1@njxzc.edu.cn for consultation.

If you have understood the above content and are willing to participate in this research, please check the box for informed consent below and sign it.

Agree □ Disagree □


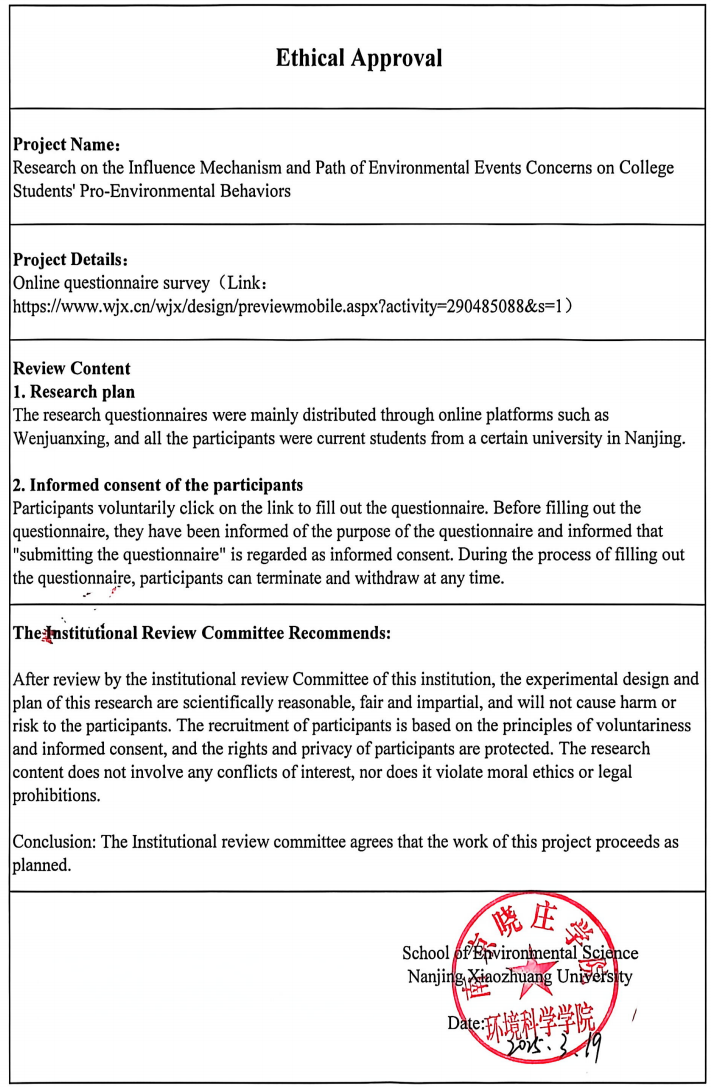

Supplement: Supplementary file 1 [file Table_1.doc]
